# Predictive validity of two process-of-care quality measures for residential substance use disorder treatment

**DOI:** 10.1186/s13722-015-0042-5

**Published:** 2015-10-31

**Authors:** Alex H. S. Harris, Shalini Gupta, Thomas Bowe, Laura S. Ellerbe, Tyler E. Phelps, Anna D. Rubinsky, John W. Finney, Steven M. Asch, Keith Humphreys, Jodie Trafton

**Affiliations:** Center for Innovation to Implementation, Health Services Research and Development Service, VA Palo Alto Health Care System, Menlo Park, CA USA

**Keywords:** Quality measurement, Residential treatment, Continuing care, Retension

## Abstract

**Background:**

In order to monitor and ultimately improve the quality of addiction treatment, professional societies, health care systems, and addiction treatment programs must establish clinical practice standards and then operationalize these standards into reliable, valid, and feasible quality measures. Before being implemented, quality measures should undergo tests of validity, including predictive validity. Predictive validity refers to the association between process-of-care quality measures and subsequent patient outcomes. This study evaluated the predictive validity of two process quality measures of residential substance use disorder (SUD) treatment.

**Methods:**

Washington Circle (WC) Continuity of Care quality measure is the proportion of patients having an outpatient SUD treatment encounter within 14 days after discharge from residential SUD treatment. The Early Discharge measure is the proportion of patients admitted to residential SUD treatment who discharged within 1 week of admission. The predictive validity of these process measures was evaluated in US Veterans Health Administration patients for whom utilization-based outcome and 2-year mortality data were available. Propensity score-weighted, mixed effects regression adjusted for pre-index imbalances between patients who did and did not meet the measures’ criteria and clustering of patients within facilities.

**Results:**

For the WC Continuity of Care measure, 76 % of 10,064 patients had a follow-up visit within 14 days of discharge. In propensity score-weighted models, patients who had a follow-up visit had a lower 2-year mortality rate [odds ratio (OR) = 0.77, p = 0.008], but no difference in subsequent detoxification episodes relative to patients without a follow-up visit. For the Early Discharge measure, 9.6 % of 10,176 discharged early and had significantly higher 2-year mortality (OR = 1.49, p < 0.001) and more subsequent detoxification episodes.

**Conclusions:**

These two measures of residential SUD treatment quality have strong associations with 2-year mortality and the Early Discharge measure is also associated with more subsequent detoxification episodes. These results provide initial support for the predictive validity of residential SUD treatment quality measures and represent the first time that any SUD quality measure has been shown to predict subsequent mortality.

## Background

In order to monitor and ultimately improve the quality of addiction treatment, professional organizations, health care systems, and addiction treatment programs must establish clinical practice standards and then operationalize these standards into reliable, valid, and feasible quality measures. Quality measures can help define and motivate guideline-congruent care, reveal gaps in the continuum of care, identify poor performers to whom quality improvement efforts can be targeted, and may provide an empirically-justified basis for performance-based incentive programs. However, these benefits can only be realized if quality measures are valid and avoid unintended consequences, neither of which is a given [1, 2].

Improvement in clinically important outcomes is the most prominent goal of health care and therefore may seem like the only legitimate “gold standard” for measuring treatment quality. But using outcomes alone as quality measures presents at least three difficulties. First, post-treatment outcome data are often expensive and logistically difficult to collect. Second, using outcomes directly as quality measures requires risk adjustments for casemix and making debatable assumptions regarding missing baseline and follow-up data [3]. Third, outcome data alone cannot indicate if treatment access is sufficient or what structural and process changes might be needed to improve outcomes. Therefore, identifying treatment structure, access, and process measures in administrative data that reliably predict long-term outcomes is often the best available quality assessment method.

Predictive validity refers to the association between antecedent quality indicators (e.g., structural, access, and process-of-care measures) and subsequent quality indicators, such as patient outcomes. Associations between process-of-care measures and outcomes can be driven by many factors, including the fidelity with which the process can be operationalized with available data [4], selection of an appropriate outcome, the temporal proximity of the outcome to the clinical process, limited power to detect small effects, and potential bias due to unmeasured confounding factors [5]. Therefore, care and humility are indicated when interpreting the results of predictive validity studies. Regardless, if the presumed association between a process-of-care quality measure and patient outcomes cannot be demonstrated, the measure’s validity should be considered suspect.

Formulating and implementing quality measures without careful empirical validation exposes all stakeholders to many risks, including promoting poor or incomplete care, and diverting effort and attention from potentially more important activities. Measures are often specified and implemented before enough data and/or interest accumulates to conduct validation studies. In diverse areas of medicine, even when careful validation is undertaken, the expected associations between process-of-care measures and outcomes are often absent or weaker than expected. Furthermore, when previously validated measures are put into use, particularly with incentives, changes in care or coding practices can lead to changes in predictive validity or other unintended consequences [6–10]. Addiction treatment quality measures have not been immune from these challenges [2, 11–15].

Residential addiction treatment programs are important features of the continuum of care for patients with SUD. As an intensive, relatively costly setting for SUD treatment, these programs are usually reserved for patients who need high levels of support, supervision, and stabilization before transitioning to outpatient continuing care. Two process-of-care measures have been proposed to capture elements of residential addiction program quality, one proposed by the Washington Circle and one by Rand and the Altarum Institute. The Washington Circle (WC) was a group of national experts that sought to improve the accessibility and effectiveness of SUD treatment through the use of quality measurement systems. One of the WC’s major activities was developing quality measures for SUD treatment for publicly-funded and commercially-insured systems of care. One WC measure focuses on outpatient continuing care after residential treatment [16]. WC Continuity of Care is a dichotomous measure indicating whether or not a patient had an outpatient SUD treatment encounter within 14 days after discharge from residential treatment. The measure is intended to assess the minimum necessary service to provide quality treatment, but not necessarily optimal or sufficient continuity of care after residential treatment.

Garner et al. [17] evaluated the predictive validity of the WC Continuity of Care measure in a sample of 342 adolescents in long-term residential addiction treatment who were randomly assigned at discharge to either standard continuing care or an assertive continuing care condition. Overall, adolescent patients whose treatment met the WC Continuity of Care criterion were significantly more likely to have achieved recovery status (no alcohol or other drug use, abuse, or dependence symptoms while living in the community during the previous 30 days) at 3 months (OR = 1.92, p < 0.05). However, the predictive validity of this measure has never been evaluated in adult patients or beyond 3 months.

The other quality measure pertaining to residential SUD treatment was developed by RAND and the Altarum Institute as part of a national evaluation of mental health services commissioned by the US Veterans Health Administration (VHA). The measure taps the proportion of patients with SUD diagnoses admitted to residential SUD treatment who are discharged or self-discharge within 1 week of admission (“Early Discharge”). Patients who discharge early from residential addiction treatment programs typically have poorer outcomes and are more likely to be readmitted [18]. However, the strength of evidence for these associations is modest and the predictive validity of the Early Discharge quality measure itself has never been evaluated.

Therefore, the purpose of this study was to examine the predictive validity of these two process-of-care quality measures in the VHA, a large integrated health care system with extensive SUD treatment services. Because we lack data on more clinically detailed outcomes, such as improvements in symptoms and functioning, the primary outcomes in this study are 2-year mortality and patterns of utilization that might signify either successful stabilization in an outpatient setting or an escalation of symptoms requiring subsequent readmission or detoxification. Specifically, we hypothesized that WC Continuity of Care would be positively associated with subsequent outpatient SUD and mental health treatment after the 14-day post-discharge observation period and negatively associated with subsequent SUD and psychiatric inpatient admissions, detoxification episodes, as well as 2-year mortality. We hypothesized that these relationships would be in the opposite direction for the Early Discharge measure. If evidence can be found for the predictive validity of these measures, they could be used for system monitoring and quality improvement purposes in VHA’s Mental Health Information System [19], or by other integrated health care systems.

## Methods

### Calculating the Continuity of Care measure

Using VHA’s National Patient Care Database, we identified all patients with an SUD diagnosis who discharged to the community from one of VHA’s 54 Substance Abuse Residential Rehabilitation Programs (SARRTPs; bed section codes: 27, 37, 85, 86, 88, 111) in fiscal year 2009 (FY09). We then determined whether each patient had an outpatient SUD visit within 14 days of discharge. SUD outpatient visits were defined as a clinical encounter in an SUD or mental health clinic in which an SUD diagnosis was recorded. Patients who transferred from the SARRTP to another residential setting were excluded from the denominator.

### Calculating the Early Discharge measure

Using VHA’s National Patient Care Database, we identified all patients with an admission to one of VHA’s SARRTPs in FY09 with a SUD diagnosis, and determined whether each patient was discharged from the program to the community within 7 days. Although the denominators for the two measures are different (patients with admissions vs. patients with discharges), the samples largely overlap.

### Outcomes

We examined if each measure was associated with the following utilization-based outcomes in the 6 months after each patient either met or did not meet the measures’ criteria: Number of subsequent admissions to a SARRTP, number of admissions to other mental health residential or inpatient programs, number of outpatient encounters in SUD clinics, number of outpatient encounters in mental health clinics, and number of detoxification episodes. We also examined the association of each measure with 2-year mortality as determined by the VHA Vital Status file.

### Propensity score modeling

Estimation of process-outcome associations from observational data is difficult because exposure to the process of care is non-random and often confounded with patient or program characteristics. Propensity score methods are often used to adjust for confounding. Here, a propensity score is defined as the probability of meeting a performance measure conditional on pre-treatment covariates. Several methods have been developed to calculate propensity scores. We used boosted regression to estimate each patient’s propensity score for meeting each process-of-care measure. Boosted regression has been shown to produce models with less prediction error than other common methods [20]. One-year pre-index variables used in the propensity score models were age, gender, marital status (Y/N), race/ethnicity, co-morbid psychiatric diagnoses, traumatic brain injury, co-morbid medical diagnoses (e.g., diabetes, hepatitis C), homelessness, outpatient and residential SUD and mental health service utilization, and number of prior detoxification episodes.

### Modeling associations between process measures and outcomes

For each process-of-care quality measure, using a propensity-score weighted [20], mixed-effects regression model, we evaluated if getting the measure-specified care (yes/no) was associated with patient-level outcomes beyond the possible effect of facility-level performance on the measure [21]. These models included VHA facility as a random effect and an exchangeable covariance structure. Utilization-based outcomes were modeled by mixed-effects, zero-inflated, negative binomial regression models. Two-year mortality was modeled in mixed-effects logistic regression models.

### Facility-level variability

In order to assess the extent to which performance on these measures varied between facilities, we calculated descriptive statistics and histograms of the distributions of facility-level performance of the VHA facilities with residential SUD programs.

## Results

For the WC Continuity of Care measure, 10,064 patients met the denominator criterion (i.e., discharged from a residential SUD treatment program in FY09) and 7648 (76 %) met the numerator criterion by having a follow-up visit within 14 days of discharge. The characteristics of patients who met versus did not meet the measure’s numerator criterion are presented in Table 1, along with the characteristics of the “failed to meet” sample after weighting with propensity scores. Even, before propensity score weighting, the two groups were very similar on most characteristics. The most pronounced differences between patients meeting and not meeting the numerator criterion were the number of SUD outpatient visits in the previous 6 months (15.4 vs. 9.8), homelessness in the previous year (30.1 vs. 26.7 %), and HIV positive status (1.8 vs. 2.6 %). All of these differences were reduced or eliminated by the weighting procedure.

**Table 1:** Demographic and pre-index characteristics of patients meeting and not meeting the WC Continuity of Care numerator criterion

Characteristic	Met (N = 7648)	Did not meet (N = 2416)	Did not meet, weighted
Mean age (years)	49.1	49.8	49.1
Female	4.9 %	4.1 %	4.6 %
Caucasian	56.8 %	58.1 %	57.1 %
Married	21.8 %	22.2 %	22.0 %
Homeless in previous year	30.1 %	26.7 %	30.0 %
SUD outpatient visits	15.4	9.8	13.7
MH outpatient visits	14.5	12.0	13.0
Residential SUD admissions	0.07	0.08	0.07
Detoxification episodes	0.55	0.55	0.57
Co-morbid psychiatric diagnoses	80.1 %	80.0 %	80.7 %
Traumatic brain injury	2.9 %	2.6 %	2.6 %
HIV positive status	1.8 %	2.6 %	1.8 %

Separate co-morbid medical diagnoses were included in the model but not presented in this table. No notable differences existed in these variables

**Fig. 1:**
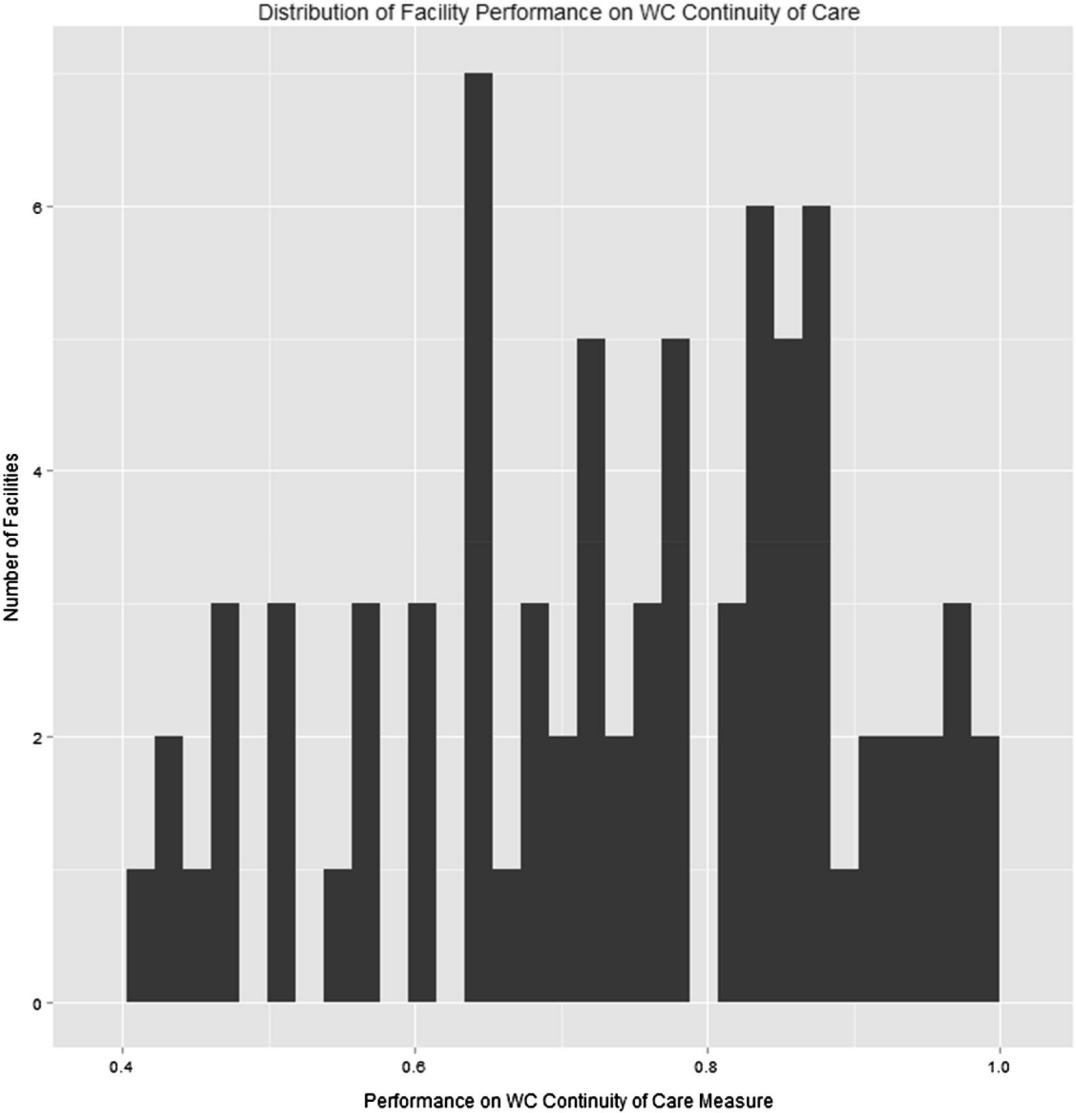
Distribution of facility performance on WC Continuity of Care

In propensity score-weighted models, patients whose care met the WC Continuity of Care measure had significantly more subsequent mental health admissions (b = 0.15, p = 0.015), significantly more mental health outpatient visits (b = 0.42, p < 0.001), and significantly more SUD outpatient visits (b = 0.65, p < 0.001). The measure was not significantly associated with number of subsequent admissions to residential SUD treatment programs or number of detoxification episodes. Notably, the odds of death in the two post-discharge years was significantly lower for patients who received the measure-specified continuity of care (4.35 vs. 5.57 %; OR = 0.77, p = 0.008). The unadjusted and propensity score-adjusted group differences in 2-year mortality and mean number of detoxifications in 6 months are presented in Table 2. Also, Fig. 1 shows substantial facility-level variation (41–99 %) in the WC Continuity of Care measure.

**Table 2:** Unadjusted and adjusted outcomes in patients meeting and not meeting measure criteria

Measure	Outcome	Patients meeting criteria	Patients not meeting criteria
WC Continuity of Care	Unadjusted 2-year mortality	4.50 %	5.92 %
	Adjusted 2-year mortality	4.35 %	5.57 %
	Unadjusted mean detoxes in 6 months	0.171	0.177
	Adjusted mean detoxes in 6 months	0.175	0.185
Early Discharge	Unadjusted 2-year mortality	7.30 %	4.72 %
	Adjusted 2-year mortality	6.29 %	4.29 %
	Unadjusted mean detoxes in 6 months	0.350	0.225
	Adjusted mean detoxes in 6 months	0.240	0.215

Adjustments refer to propensity score weighting in mixed effects regression models

For the Early Discharge measure, 10,176 patients met the denominator criterion (i.e., admitted to a residential SUD treatment program in FY09) and 974 (9.6 %) met the numerator criterion by experiencing discharge within 7 days. The characteristics of patients who met versus did not meet the measure’s numerator criterion are presented in Table 3, along with the characteristics of the “did not meet” sample after weighting with propensity scores. Unlike the WC Continuity of Care measure, it is considered undesirable to meet the numerator criterion for the Early Discharge measure. Again, even before propensity score weighting, the two groups were very similar on most characteristics. The most pronounced differences between patients meeting and not meeting the numerator criterion were in the number of mental health outpatient visits in the previous 6 months (15.8 vs. 13.8), Caucasian race (66.8 vs. 59.3 %), traumatic brain injury (4.3 vs. 2.7 %), and HIV positive status (2.7 vs. 1.8 %). All of these differences were reduced or eliminated by the weighting procedure.

**Table 3:** Demographic and pre-index characteristics of patients meeting and not meeting the Early Discharge numerator criterion

Characteristic	Met (N = 974)	Did not meet (N = 9202)	Did not meet, weighted
Mean age (years)	49.2	49.4	49.1
Female	5.7 %	4.5 %	5.3 %
Caucasian	66.8 %	59.3 %	65.6 %
Married	24.3 %	22.1 %	23.8 %
Homeless in previous year	29.2 %	29.4 %	29.6 %
SUD outpatient visits	15.8	14.1	15.4
MH outpatient visits	15.8	13.8	15.5
Residential SUD admissions	0.09	0.07	0.08
Detoxification episodes	0.72	0.54	0.70
Co-morbid psychiatric diagnoses	85.0 %	80.7 %	84.5 %
Traumatic brain injury	4.3 %	2.7 %	4.0 %
HIV positive status	2.7 %	1.8 %	2.2 %

Separate co-morbid medical diagnoses were included in the model but not presented in this table. No notable differences existed in these variables

**Fig. 2:**
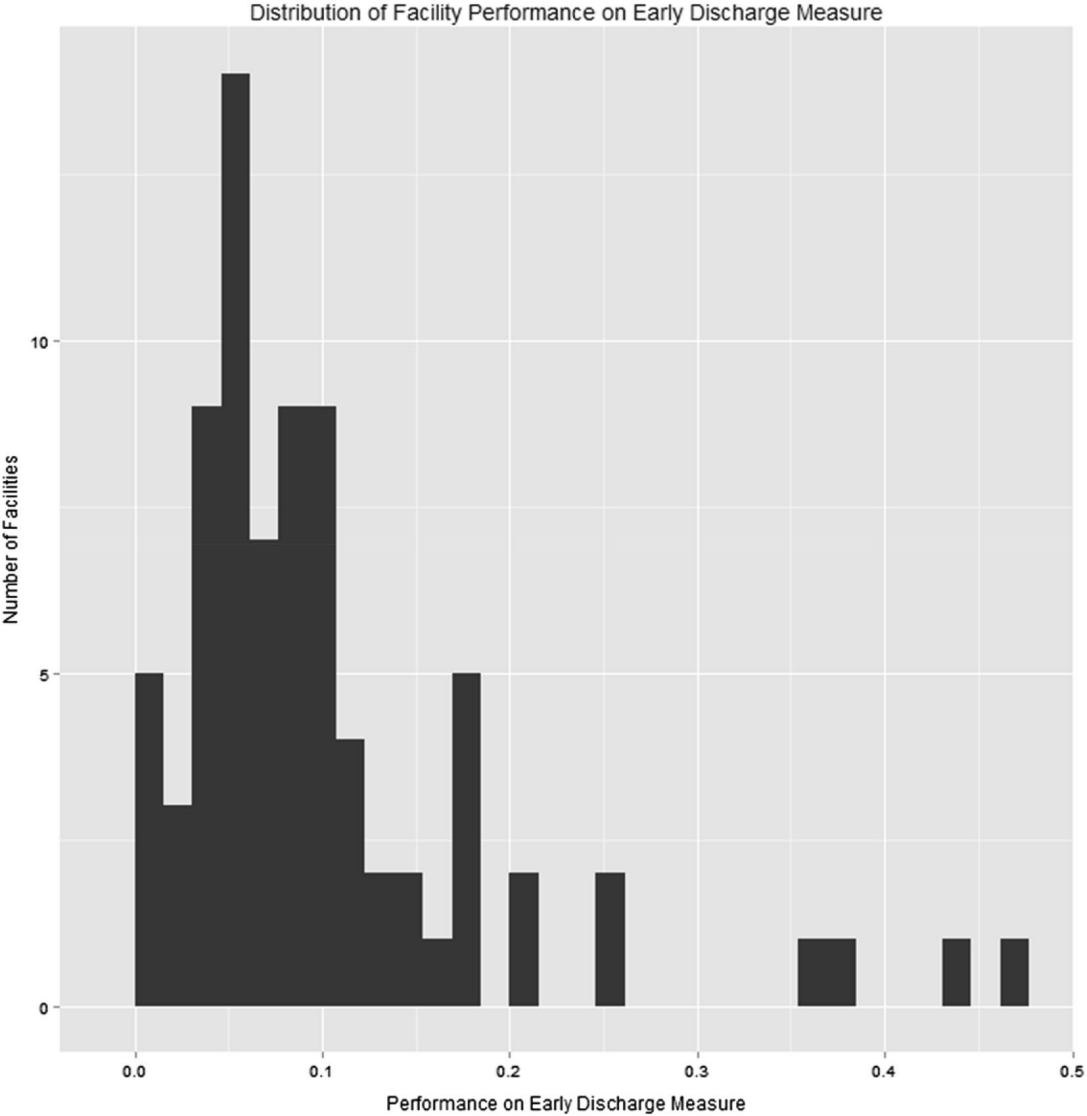
Distribution of facility performance on Early Discharge measure

In propensity score-weighted models, patients who met the numerator criterion for early discharge had significantly fewer mental health admissions (*b* = −0.65, p < 0.001), mental health outpatient visits (*b* = −0.50, p < 0.001), SUD outpatient visits (*b* = −0.88, p < 0.001), and admissions to residential SUD treatment programs (*b* = −1.05, p < 0.001) in the following 6 months. Patients discharging early also had significantly more subsequent detoxification episodes (*b* = 0.11, p = 0.037). Finally, the odds of death in the two post-discharge years was significantly higher in patients who discharged early and thereby met the numerator criterion (7.30 vs. 4.72 %; OR = 1.49, p < 0.001). The unadjusted and propensity score-adjusted group differences in 2-year mortality and mean number of detoxifications in 6 months are presented in Table 2. Also, Fig. 2 shows substantial facility-level variation in this measure from 0 to 46 %.

## Discussion

In this study, we examined the predictive validity of two process-of-care quality measures for residential SUD treatment in a large sample of patients in a very large integrated health care system. Ideally, data would have been available to examine the associations of these measures with near- and long-term changes in symptoms and functioning, instead of only administrative and utilization data. We originally had hypotheses about the relationships between these measures and patterns of utilization, such as outpatient SUD treatment engagement, that might signify successful stabilization, and patterns of utilization, such as subsequent readmission or detoxification, that might signify an escalation. However, the results indicated that more utilization specified in the measures was generally associated with more subsequent utilization of both types. In other words, markers of engagement in the health care system are associated with other markers of later engagement in the health care system. In retrospect, this is unsurprising and consistent with research in other clinical areas. The one type of utilization that ran counter to this general trend, however, was subsequent detoxification episodes. Although the WC Continuity of Care measure was unrelated to subsequent detoxification episodes, the Early Discharge measure (i.e., less initial utilization) was associated with significantly more subsequent detoxification episodes.

Even more impressive, both measures were associated with risk of death in the two post-discharge years in the expected directions: Continuity of Care with lower risk of death and Early Discharge with higher risk of death. These results are noteworthy given that mortality is a conceptually distal outcome of SUD treatment relative to, say, substance use. Previous efforts in the area of addiction and more broadly to examine associations between continuity of care and retention quality measures and outcomes have largely found none (e.g., [22–24]) with some exceptions (e.g., [17, 25, 26]). However, this is the first study of addiction treatment process measures to find associations with risk of death. It is possible but unlikely that the associations between these measures and risk of death are directly causal. Perhaps more likely is that these processes facilitate or are proxies for different levels of engagement with the health care system, which in turn affect mortality risk. Alternatively, these processes may be associated with unmeasured factors that are linked to mortality. In an event, finding SUD treatment process-of-care quality measures that are so strongly linked with subsequent risk of death is remarkable.

This study has several limitations. First, the VHA is a large integrated health care system serving US military Veterans. It is unknown to what extent these findings generalize to different health care systems or settings. Second, although patients who met the measures and patients who did not meet the measures were very similar on measured characteristics, and differences that did exist were trivial after propensity score weighting, these patients may have also differed on unmeasured characteristics, such as SUD severity or travel time to care, that could affect outcomes.

With these caveats in mind, it is still noteworthy that these two process-of-care quality measures for residential SUD treatment are linked to 2-year mortality risk, and the Early Discharge measure was associated with subsequent detoxification episodes. These results provide important new evidence supporting the predictive validity of the WC Continuity of Care measure and the Early Discharge measure, and thereby, affirming their value as tools to monitor the quality of residential SUD care for adults. Because these results were not guaranteed, we believe our study shows the importance of subjecting quality measures to validity checks before implementing them in clinical practice. Where these measures are implemented, especially if attached to incentives or consequences for providers or systems of care, it is critical to make sure that they do not drive unintended changes in practice (e.g., instituting a cursory follow-up program to meet the measure) that will dilute or destroy the process-outcome associations found in this study.

## Abbreviations

SUD: substance use disorder
WC: Washington Circle
VHA: US Veterans Health Administration
SARRTP: Substance Abuse Residential Rehabilitation Programs
FY: fiscal year
OR: odds ratio
